# Global warming modifies the seasonal distribution of clutches on a Mediterranean great tit population

**DOI:** 10.1007/s00484-022-02415-x

**Published:** 2022-12-12

**Authors:** Iris Solís, Elena Álvarez, Emilio Barba

**Affiliations:** Cavanilles Institute of Biodiversity and Evolutionary Biology, University of Valencia, C/ Catedrático José, Beltrán 2, 46980 Paterna, Spain

**Keywords:** Breeding season length, Climate change, Phenology, *Parus major*, Spain, Spring temperatures

## Abstract

Global warming has multiple effects on phenological events on a wide range of plants and animals. Specifically, many bird species have advanced the start of their breeding season, which could also imply an extension in its duration and also a change in the distribution of clutches throughout the breeding season. We have tested whether this occurred in a population of Great Tits (*Parus major*), in Sagunto (eastern Spain). The increase of March temperatures between 1986 and 2019 was related to an advancement of the breeding season. Although the breeding season was longer in years with higher June temperatures, the length did not show a temporal trend throughout the study period. The clutches were more concentrated at the beginning of the season (increase in the skewness), while the kurtosis (“tailedness” of the distribution) or the modality did not change significantly. Finally, the number of “equally good months” for breeding (a combined measure of length and distribution) has not changed throughout the years. Overall, all these phenological changes probably caused the observed increased proportion of pairs laying two clutches per year. It remains to be studied whether this increase in reproductive effort has positive or negative impact on fitness.

## Introduction

Global mean temperatures have been increasing throughout the last decades, and the rate of warming is accelerating in the last years (IPCC 2019). This global warming has been shown to have multiple effects on environmental conditions and biodiversity, and one of the most frequently reported is an advance of some phenological events on a wide range of plants and animals (Gordo & Sanz 2005; Thackeray et al. 2016; Halupka et al. 2020). In the case of birds, the clearest pattern is the advancement of the breeding period in both migratory and resident species, although it seems to be more fixed in the latter (Tryjanowski et al. 2004; Parmesan 2007; Hällfors et al. 2020; Bates et al. 2022).

An interesting potential consequence, which has received little attention to date, is whether this advancement of the start of the breeding season implies changes in the duration of the breeding season. Menzel and Fabian (1999) showed that spring events in Europe have advanced a mean of 6 days, while autumn events have delayed by 4.8 days between 1959 and 1993, therefore increasing the duration of the growing season by 10.8 days over this period. This suggests that also the duration of the birds’ breeding season could be extended, by both advancing its start and delaying its termination. The few results available to date, however, do not support this expectation. For example, Møller et al. (2010), in Denmark, found that, while the breeding season of the wood pigeon *Columba palumbus* has increased by 36 days (48%) over the period 1970–2007, that of the sandwich tern *Sterna sandvicensis* has contracted by 36 days (70%) during the same period. This, and several other studies (e.g. Visser et al. 2003; Najmanová & Adamík, 2009; Gullett et al. 2013; Jankowiak et al. 2014; Halupka and Halupka 2017; Hällfors et al. 2020), show that although the potential magnitude of the changes in the duration of the breeding season might be large, the outcome (increase or decrease) might differ between regions, species, and probably between populations of the same species. Apart from the characteristics of the particular species or population (e.g., number of clutches per season, migratory tendency), details as when exactly and how much temperatures change, and how this affects abundance and seasonal distribution of food resources for a particular species, could determine the changes in the length of the breeding season and the distribution of clutches throughout it.

Apart from the interval of days in which clutches are laid, the distribution of clutches within this interval has an outmost importance for evolutionary processes (Møller et al., 2010). Apart from the work of some authors, as Laaksonen et al. (2006) or Goodenough et al. (2011), who have considered temporal changes in skewness or kurtosis of the distribution of clutches, this issue has been mostly overlooked in the literature.

The question of the duration of the breeding season, along with the seasonal distribution of the clutches, was tackled by Wyndham (1986). He used a measure, called “Equally Good Months” for breeding (EGM hereafter), which was derived from a diversity index and took into account the number of broods initiated each month (see index formula in “Methods”). Considering not only when the season starts and finishes, but also the distribution of clutches throughout it, this measure is a combined estimator of both the time window for breeding and the “goodness” of the available period. Thus, given the same dates of start and termination, the index would be higher if the clutches are evenly distributed throughout the period, and lower if they are mostly concentrated in a small period with only a few ones being laid early and/or late. This approach has been successfully used to explore spatial differences in the length of the breeding seasons (Wyndham 1986; Yom-Tov 1987; Dean et al. 2009) but, to our knowledge, it has not been used to explore temporal changes and, more explicitly, temporal changes related to climate warming.

Summarizing, several studies have addressed how the dates of start and termination of the breeding season, the length of the breeding season, and the distribution of the clutches throughout it have changed over the years in relation to changes in temperature. These studies have been performed for different species, over diverse time periods, using different methodologies, and considering one or few of the above parameters, sometimes including only first clutches. But, to our knowledge, there has not been an integrating approach, considering all the facets of the question of how climate change is related to phenological changes in the breeding attempts of birds over the whole breeding season.

The great tit is a small passerine for which a huge amount of information on laying dates has been collected in many European populations for decades (e.g., Bailey et al. 2022). Visser et al. (2003) examined the changes in mean laying dates of 13 European great tit populations, and they found that only five of them had advanced their breeding seasons. Changes in the distribution of first clutches have been examined in a British population, finding an increase of the skewness (Goodenough et al. 2011). However, to the best of our knowledge, there is no explicit information about the existence of changes in the duration of the breeding season, nor about temporal changes in the distribution of clutches throughout the whole breeding season. Based on data collected about the breeding phenology of a great tit population in Eastern Spain over 34 years, we aim to answer the following questions: (1) Have there been temporal trends in spring temperatures over these 34 years? (2) Have there been temporal changes in the start, termination, duration of the breeding season, and in the seasonal distribution of the clutches throughout this period? (3) If so, were the observed changes in these phenological events related to changes in temperatures?

## Materials and methods

### Study area and general field methods

The study was conducted in a great tit population breeding in nestboxes in eastern Spain throughout 34 years (1986–2019). The study area was located within an extensive orange *Citrus aurantium* monoculture plantation, near Sagunto and close to the Mediterranean Sea (39° 42′ N, 0° 15′ W, 30 m a.s.l.), in the Valencian Community. The study area has increased over the years, from about 150 ha in the first years to about 450 in the recent ones, but the density of nestboxes has remained almost constant at about 1 nestbox per ha. Roughly, the number of pairs each year is related to the extent of the area with nestboxes.

Each year, wooden nestboxes are hanged on the orange trees by late February, and they are removed after the breeding season, by late July. As far as possible, nestboxes are placed in the same tree each year. Each nestbox was checked at least weekly to find new nests and daily in some periods to record basic breeding parameters (e.g., Rodríguez & Barba 2016; Solís et al. 2021). Regular checking of all the nestboxes was terminated when no new nests appeared in two consecutive weeks after middle June, so we are confident that all the breeding attempts of each season were recorded.

For this study, one of the most important parameters was the date of laying of the first egg. Given the pattern of visits, considering that the mean clutch size of the population was 8 eggs (Atiénzar et al. 2016) , and that clutches with less than 7 eggs were scarce, the date of laying of the first egg of most clutches could be back-calculated assuming the laying of one egg per day. In the relatively few cases when the clutch was found complete, we additionally assumed an incubation period of 13 days and that incubation started with the laying of the last egg (Álvarez & Barba 2014) to calculate the date of laying of the first egg. Dates are presented as “April date” (day 1 = 1st April).

Each year, the breeding population at the study area was estimated as the number of first clutches laid. We considered as first clutches those started within the first 30 days after the start of the first clutch of that year (van Noordwijk et al. 1995; Visser et al. 2003; Álvarez & Barba 2014). The rest of the clutches, including second, replacement, and unknown ones (see, e.g., Solís et al. 2021 for details), were grouped together as “late clutches.” The percentage of late clutches was independent from the number of breeding pairs (*R*^2^ = 2.2%, *F*_1,26_ = 0.574, *P* = 0.456).

### Phenological indices

In our study area, great tits usually start building nests around middle March, start laying their first clutches between very late March and late April, and late clutches are laid from late April to early June (Atiénzar et al. 2016) . In the present dataset, from a total of 4237 clutches studied, only 77 clutches (1,8%) were initiated in March, being the earliest laying date the 24th of March (years 2001 and 2012), while 232 clutches (5,5%) were initiated in June, the last one being started on 27 June (year 1988).

For each breeding season, we estimated the following phenological indices:

1. Mean laying date of first clutches: the average date of laying of the first egg for all first clutches of that year. This is the measure used to estimate the beginning of the breeding season in most studies (Vaugoyeau et al., 2016, and references therein), and it is highly correlated with median laying dates used in other ones (e.g., Gullett et al. 2013).
2. Median laying date of all the clutches: the median date of laying of the first egg for all the clutches recorded in that year. In this case, we used the median to give a more accurate idea of the center of the distribution of the clutches throughout the season, although in our dataset, mean and median dates were closely related (*R*^2^ = 78.4%, *F*_1,33_ = 116.28, *P* < 0.001).
3. Date of laying initiation: 10th percentile of the clutches started in that year. This is a commonly used index (e.g., Møller et al. 2010; Gullett et al. 2013) and avoids the potential disproportionate effects of very early clutches.
4. Date of laying termination: 90th percentile of all the clutches laid in that year, used by the same reasons outlined above.
5. Duration of the breeding season: interval between the 10th and 90th percentile of all known clutches of that year (e.g., Møller et al. 2010; Gullett et al. 2013).
6. Number of EGM: index of the distribution of all the clutches laid throughout the whole season. EGM for each year was calculated as EGM = exp (− ∑ *p*_*i*_ ln *p*_*i*_), where *p*_*i*_ is the number of nests initiated in each month from March to June (Macthur 1964; Wyndham 1986) . To make this analysis more sensitive, we also considered periods of 15 days during the breeding season, starting on the 15th of March, therefore having 7 “fortnights” instead of 4 months. The results were the same as using whole months, so the latter are not shown.

We also searched for temporal autocorrelation in the residuals of these indices with Durbin-Watson’s test. We found a slightly positive temporal autocorrelation, but these values vary between 1.51 and 2.04, so we do not think it is a factor of major concern for the reliability of our results.

### Temperature data

We collected data from mean daily temperatures from February to June of each of the 34 study years from the meteorological station “Sagunto-Pontazgo,” placed 4 km from the study area. From these, mean monthly temperatures were calculated. We used monthly temperatures to explicitly test for the effects of temperatures in each particular month on the variables of interest. For example, temperatures in May or June are not expected to affect the start of the breeding season occurring in March–April.

### Data analysis

We first explored the existence of temporal trends in mean monthly spring temperature and in phenological indices, using year as a predictor, with regression models. We tried linear and quadratic regressions, but linear ones fitted to the data better and only these are presented. We also used Pearson’s correlations to study the correlation between monthly temperatures, especially to get an idea of how temperatures during the breeding season could be predicted by those in early spring. After this, to investigate the effects of ambient temperatures on reproductive phenology, and considering the dates of laying in our study area (see above), we modeled (a) mean laying date in response to temperatures during February, March, and April; (b) date of laying initiation in response to temperatures during February, March, and April; (c) median laying date of all the clutches in response to temperatures during February, March, April, May, and June; (d) proportion of late clutches (over the number of breeding pairs) in response to mean laying date of the first clutches and to temperature during April, May, and June; (e) date of laying termination in response to date of laying initiation and temperatures during April, May, and June; (f) duration of the breeding season in response to date of laying initiation, date of laying termination, and temperatures during February, March, April, May, and June; and (g) EGM in response to date of laying initiation, date of laying termination, and temperatures during February, March, April, May, and June. In all cases, we explored the fit of linear and quadratic regressions, but linear ones describe better the data in all cases, so quadratic ones are not presented.

Laying date distribution parameters (kurtosis, skewness and modality) were also quantified and their potential temporal changes examined for all clutches. Kurtosis [distributions becoming more platykurtic (flattened) or leptokurtic (peaked)] values were regressed against year. Skewness values were standardized to create z-scores by dividing skewness by the standard error of skewness (Field 2000), and z-scores were regressed against year. Skewness of a normal distribution has value 0; a right-skewed distribution with a long tail of values greater than the mean has values > 0, and, on the contrary, a left-skewed distribution has values < 0. Finally, to test for changes in modality (distribution becoming more bi- or multi-modal), annual Hartigan’s dip test statistic (Hartigan & Hartigan 1985) was used, regressing it against year. A positive correlation between annual dip test scores and year would indicate an increasing tendency to bi- or multi-modality. As above, linear models described better the data than quadratic ones.

All analyses were conducted with IBM SPSS Statistics 25 software.

## Results

### Temperature variation

**Fig. 1:**
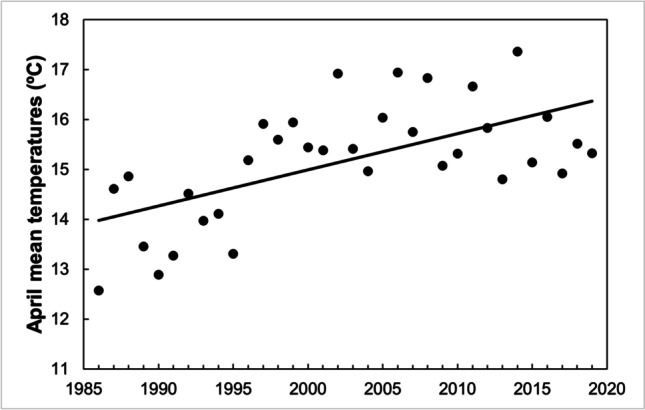
Spring temperatures increased in Sagunto from 1986 to 2019; the figure shows those of April as an example

In our study area, spring temperatures have increased significantly from 1986 to 2019 (Table 1; Fig. 1). This was so for March (+ 1.6 °C), April (+ 2.3 °C), May (+ 2.5 °C), and June (+ 2.8 °C). Mean temperatures did not increase significantly in February. It should be noted that mean temperatures in May by the end of the study period (19.5 °C in 2019, as estimated by the regression equation) were almost as hot as those of June by the start of the study period (20.6 °C in 1986). Linear regressions were used in these analyses, as they describe better the model. Pearson’s correlation revealed that mean temperature in each month was positively related with that of the previous one (Table 2). Also, June temperatures were related to April ones, so relatively hot years when the breeding season started seem to imply that they were also hot by the end of the breeding period.

**Table 1:** Linear regression results on trends on mean temperatures between 1986 and 2019 during spring months. Linear models were presented, as quadratic ones did not improve the fit in any case

Month	*R*^2^	*F*_1,33_	*P*
February	4.3%	1.436	0.24
March	18.7%	7.337	**0.011**
April	37.5%	19.212	**0.000**
May	33.2%	15.931	**0.000**
June	37.8%	19.445	**0.000**

Bold emphasis represents significant values

**Table 2:** Pearson’s correlation coefficients matrix applied to February, March, April, May, and June temperatures during the studied years (1986–2020)

	February	March	April	May	June
February	1				
March	0.381*	1			
April	0.290	0.594**	1		
May	0.286	0.259	0.578**	1	
June	0.132	0.239	0.507**	0.570**	1

^*^Correlation coefficient is significant at the 0.05 level; **Correlation coefficient is significant at the 0.01 level

Bold emphasis represents significant *P*-values

### Start, termination, and duration of the breeding season

**Fig. 2:**
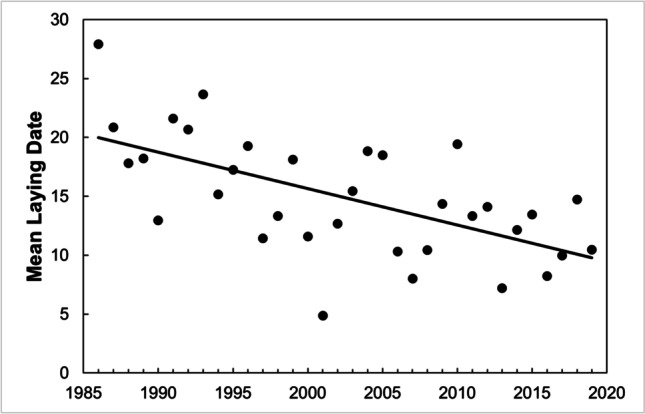
Mean laying dates of first clutches became earlier in Sagunto from 1986 to 2019. Dates presented as “April dates” (1 = 1st April)

The mean laying date of the first clutches over the study period (1986–2019) was April 15 (mean = 14.89, SD = 5.06, range = 4.87–27.91, *n* = 34 years). Mean laying date of first clutches has advanced (*R*^2^ = 37.1%, *F*_1,33_ = 18.85, *P* < 0.001) by 10.22 days over the 34 years, a mean rate of 0.30 days per year (Fig. 2). This advance was also significant if we consider the 10th percentile of the laying dates (*R*^2^ = 20.5%, *F*_1,33_ = 8.25, *P* = 0.007), but it was a bit lower (6.92 days, 0.20 days per year). Median laying dates, considering all the clutches laid throughout the season, have also shown a significant advance (*R*^2^ = 36.9%, *F*_1,33_ = 18.69, *P* < 0.001) of 13.27 days. However, we did not find a temporal change in the termination of laying (90th percentile of laying dates) (*R*^2^ = 0.1%, *F*_1,33_ = 0.02, *P* = 0.90).

**Fig. 3:**
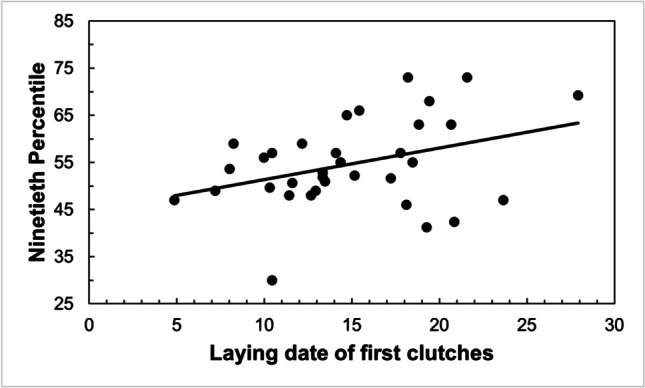
The breeding season of great tits finished earlier when the laying date of the first clutches was earlier. The end of the season was estimated as the 90th percentile of the clutch distribution

Laying date of the first clutches was earlier as February, March, and April mean temperatures increased, though the relationship was stronger with those of March (Table 3(a)). The results were virtually the same when using 10th percentile of the laying dates (Table 3(b)). Also, the median laying date of all clutches advanced as temperatures in February, March, April, May, and June were higher, being again March the month showing a stronger relationship (Table 3(c)). Finally, the breeding season finished earlier when the laying date of the first clutches was earlier (Fig. 3), and when April temperatures were higher, while there were not significant relationships with temperatures in other months (Table 3(d)).

**Table 3:** Linear regressions to test the relationship between mean temperatures and (a) mean laying dates from first clutches, (b) 10th percentile of laying dates, (c) median laying dates from all clutches and (d) 90th percentile of laying dates

Month temperatures	*R*^2^	*F*_1,33_	*P*
(a) Mean laying date from first clutches			
February	28.4%	12.681	**0.001**
March	58.2%	44.574	**0.000**
April	29.2%	13.221	**0.001**
(b) 10^th^ percentile of laying dates			
February	23.1%	9.627	**0.004**
March	42.4%	23.533	**0.000**
April	13.4%	4.933	**0.034**
(c) Median laying dates from all clutches			
February	26.7%	11.651	**0.002**
March	39.2%	20.594	**0.000**
April	31.4%	14.633	**0.001**
May	26.8%	11.728	**0.002**
June	18.1%	7.086	**0.012**
(d) 90th percentile of laying dates			
February	7.7%	2.677	0.112
March	8.4%	2.921	0.097
April	13.1%	4.814	**0.036**
May	4.0%	1.331	0.257
June	0.1%	0.024	0.878

Bold emphasis represents significant *P*-values

**Fig. 4:**
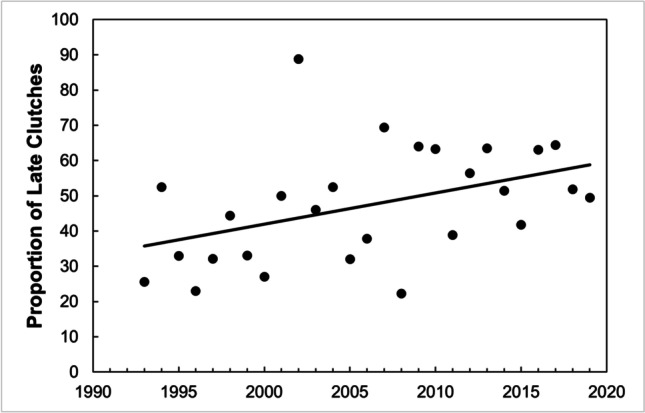
The proportion of great tit pairs that attempted a late clutch increased from 1993 to 2019

Overall, 48% of pairs from the total laid a later clutch. This proportion could be somewhat smaller if some of unknown clutches were in fact late first clutches. The proportion of late clutches increased throughout the years (linear: *R*^2^ = 18.7%, *F*_1,26_ = 5.737, *P* = 0.024; Fig. 4). We did not find any significant effect of February, March, April, May, or June’s temperature or mean laying date on the proportion of late clutches.

**Fig. 5:**
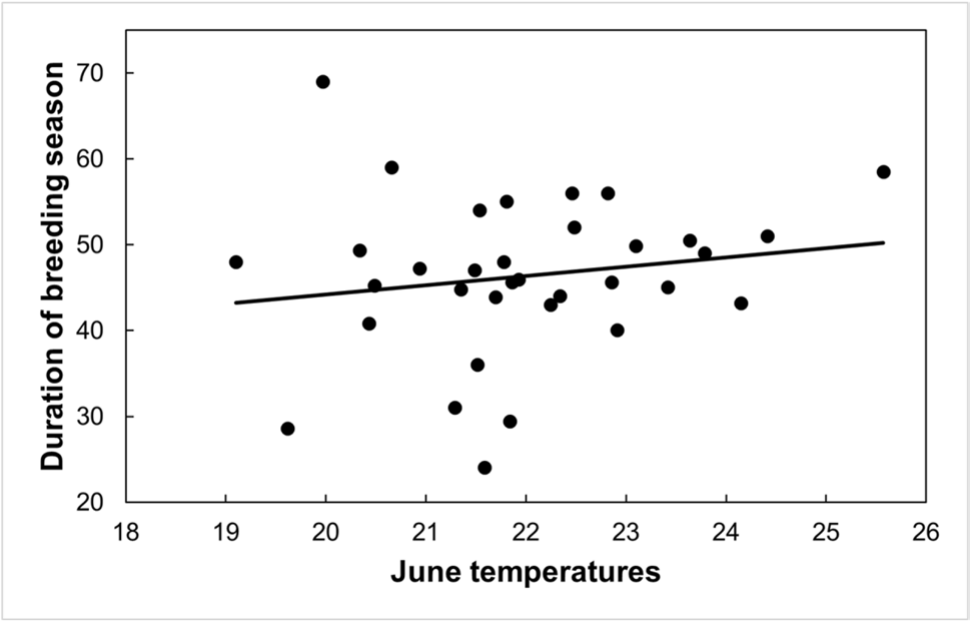
The duration of the breeding season of great tits (in days) was longer as June temperatures were higher (*R*.^2^ = 18.6%, *F*_1,26_ = 5.697, *P* = 0.025)

Considering the interval between the 10th and 90th percentiles of laying dates, the duration of the breeding season has not changed over the last 34 years (*R*^2^ = 4.1%, *F*_1,33_ = 1.374, *P* = 0.25). The duration of the breeding season was longer as June temperatures were higher (Fig. 5). We did not find any significant effect of February, March, April, or May temperatures or mean laying dates on the duration of the breeding season.

### Temporal distribution of clutches

**Fig. 6:**
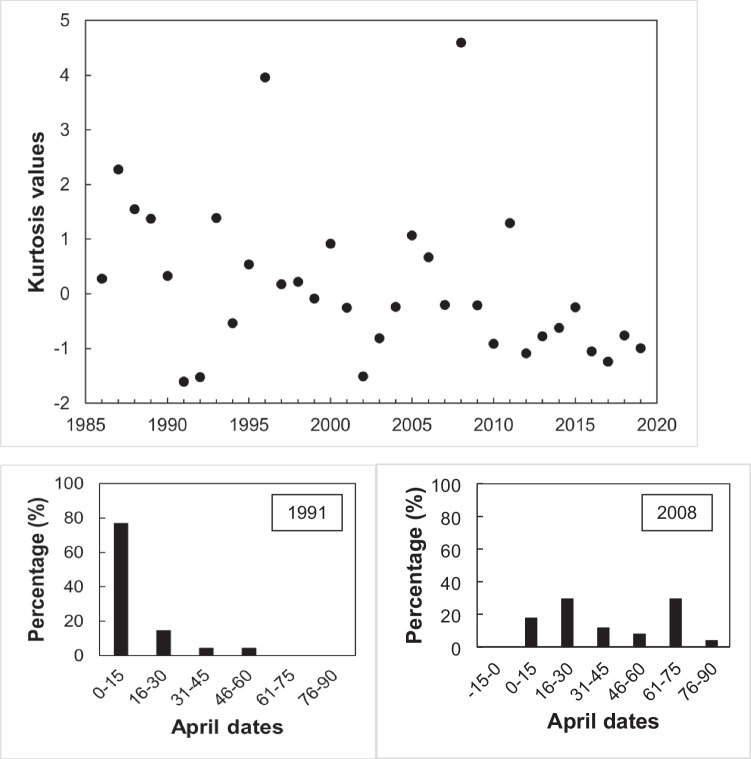
The kurtosis of the data of distribution of great tit clutches along the breeding season showed a negative trend from 1986 to 2019 with a high variation among years, although it was not statistically significative (*R*^2^ = 10.1%, *F*_1,33_ = 3.602, *P* = 0.067). Histograms show two extreme years: 1991, with the lowest kurtosis value, and 2008, with the highest one. For visual simplicity, the clutches were grouped in 15-day periods; dates are April dates

**Fig. 7:**
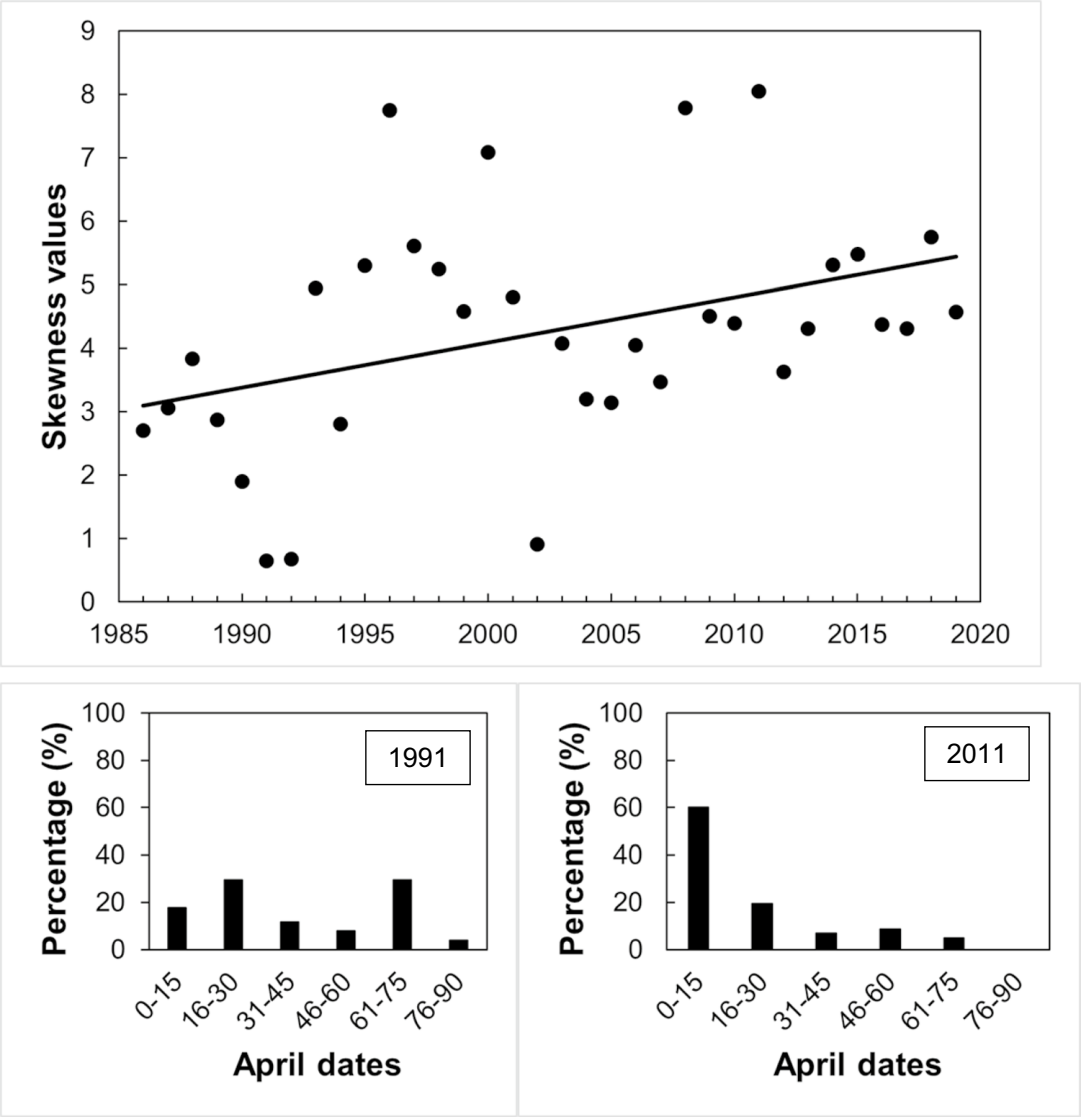
The skewness values of the distribution of great tit clutches along the breeding season increased significantly from 1986 to 2019 (*R*^2^ = 14.8%, *F*_1,33_ = 5.571, *P* = 0.025). Histograms show 2 extreme years: 1991, with the lowest skewness value, and 2011, with the highest one. For visual simplicity, the clutches were grouped in 15-day periods; dates are April dates

Annual dip test scores did not change significantly over time (*R*^2^ = 0.3%, *F*_1,33_ = 0.112, *P* = 0.74), so laying dates have not become increasingly bimodal. Kurtosis values showed a declining, though not significant, trend throughout the years (*R*^2^ = 10.1%, *F*_1,33_ = 3.602, *P* = 0.067; Fig. 6), indicating that the distribution of clutches would tend to be more platykurtic (i.e., flattened, equally distributed throughout the season). On the other hand, the skewness of the distribution increased throughout the years (*R*^2^ = 14.8%, *F*_1,33_ = 5.571, *P* = 0.025; Fig. 7), meaning that clutches are more concentrated by the beginning of the breeding season, as the distribution has become more right skewed.

Considering all the clutches each year, the number of EGMs has not changed from 1986 to 2019 (*R*^2^ = 1.2%, *F*_1,33_ = 0.377, *P* = 0.543). The number of EGM was higher when the median laying date for all the clutches was later (*R*^2^ = 36.9%, *F*_1,33_ = 18.710, *P* < 0.001), when the breeding season finished later (*R*^2^ = 45.8%, *F*_1,33_ = 27.045, *P* < 0.001), and when it was longer (*R*^2^ = 40.8%, *F*_1,33_ = 22.034, *P* < 0.001).

## Discussion

### Spring temperatures trend

Due to global warming, spring temperatures have been increasing at all spatial scales during the last decades (Visser et al. 1998; Both and Visser 2001; IPCC 2019, 2021). From the second half of the nineteenth century, global temperatures have increased significantly by 1.1 °C, and from 2016 to 2020, average temperatures were almost the highest in record. In Europe, temperatures have increased by 2 °C, and this continent has also warmed faster than any other in the recent decades (EEA 2021; IPCC 2021). In Spain, 2019 was a warm year, with a mean temperature of 15.9 °C, a value which surpasses by 0.8 °C the mean yearly value of the period 1981–2010. At a regional level, Sala et al. (2018) stated that temperatures have raised almost 1 °C in the Valencian Community from 1950 to 2016. Accordingly, we found that spring temperatures in Sagunto have increased from 1986 to 2019, especially in June, where the increase approached 3 °C. In general, temperatures in a given month were predictable from those of the previous month, which would allow appropriate decisions on reproductive investment to be made. Importantly, June temperatures were correlated with April ones, meaning that relatively warm years in early spring would also mean a hot environment in late spring and early summer.

### Advancement of the breeding season and relationship with ambient temperatures

One of the clearest effects of global warming over phenological events in birds is an advance of their breeding period (Both & Visser 2001; Both, et al. 2005; Parmesan 2007; Halupka and Halupka 2017; Hällfors et al. 2020; Halupka et al. 2020). In her meta-analysis, spanning 41 bird species from the northern hemisphere, Parmesan (2007) quantified a mean advancement of the breeding season of 3.7 days per decade. However, the period for which ambient temperatures affect the start of the breeding season differs between species and between population within species (Bailey et al. 2022), so it is worth exploring which specific temperatures are relevant for each bird population.

We have found that the mean laying date in our population of great tits in Sagunto has advanced by 10.22 days over the last 34 years. This means an advancement of 3.0 days per decade, a somewhat lower value that the mean reported by Parmesan in her study of spring phenological changes in various taxa (2007). This advancement was related to the increase of spring temperatures, especially March temperatures. Bailey et al. (2022) included the great tit population of Sagunto in their meta-analysis, concluding that the relevant time-window of temperatures was between mid-February and early April, agreeing with that reported in the present study.

### Duration of the breeding season

When studying the duration of the breeding season, it should be considered that the factors affecting the start and the termination of the breeding activities might differ, or that they might act with different intensity or in different directions. For example, temperature trends could differ in different months (e.g., Møller et al. 2010), therefore having contrasting effects on pairs starting a breeding attempt at different times of the season (e.g., Halupka et al. 2008). Also, an extended availability of resources, derived from extended growing seasons (Menzel & Fabian 1999), could promote a lengthening of the breeding season on both sides, especially in multi-brooded species (Najmanová & Adamík, 2009; Halupka & Halupka 2017). On the contrary, changes in environmental conditions could cause both a delay in the start and an advance in the termination of breeding, thus shortening the breeding season (Jankowiak et al. 2014; Hällfors et al. 2020), especially in single-brooded species in temperate regions (Najmanová & Adamík, 2009; Gullett et al. 2013; Halupka & Halupka 2017) or resident or short-migratory multi-brooded species in boreal regions (Hällfors et al., 2020). Thus, the potential magnitude of the changes in the duration of the breeding season might be large, but the outcome (increase or decrease) could not be directly predicted from increases of spring temperatures or the duration of the growing season and might differ between regions, species with different migratory strategies, and probably between populations of the same species (see, e.g., Visser et al. 2003; Tryjanowski et al. 2005; Hällfors et al. 2020).

In contrast with previous studies and previous predictions, Halupka and Halupka (2017), in their meta-analysis of changes in the length of breeding seasons of birds, with 54 northern hemisphere species (the great tit among them), showed that the duration of breeding season was not related to advances in laying dates, and instead, it was related to the degree of broodiness of the species. Thus, for multi-brooded species, the number of pairs laying more than one clutch would depend on the length of the season (e.g., Halupka et al. 2008; Halupka & Halupka 2017). In discrepancy with the latter study, Hällfors et al. (2020) could not find a relation between changes in breeding season length and broodiness, but found a relationship with migration strategies, so that only resident or short-distant migrants (which generally breed early in the season) showed a greater contraction of their breeding season.

In our study in Sagunto, with a resident and potentially multi-brooded population of great tits, the duration of the breeding season has not changed throughout the last 34 years, despite the advancement of the start of the breeding season, the increase in spring temperatures, and even the increase in the proportion of pairs attempting late clutches (Solís et al. 2021). Higher temperatures in April meant an earlier breeding season, but also an earlier end. As mentioned above, April temperatures were positively related to June temperatures, so a warm spring generally implies a hot summer, and birds seem to avoid breeding when ambient temperatures are too high. Thus, the observed increase in the length of the breeding season when June temperatures were higher should be caused mainly by an earlier start, and not so much by a lengthening of the end.

### Distribution of clutches throughout the breeding season

Simply considering the interval of days in which clutches are laid completely ignores the distribution of clutches within this interval, something that has an outmost importance for evolutionary processes (Møller et al. 2010; Goodenough et al. 2011). It has been frequently assumed that changes in the duration of the breeding season do not alter the distribution of clutches within the season, but Gullett et al. (2013) showed that this has not to be so. They showed that different outcomes could theoretically arise from similar advances, and even that the distribution of clutches throughout the season could change without changing the mean laying date (see their Fig. 2).

Although some authors have considered some measures of the distribution of clutches other than means (e.g., skewness or kurtosis) in their analyses (Laaksonen et al. 2006; Goodenough et al. 2011), they only have taken into account first clutches. Even for similar species in the same study area and years, Goodenough et al. (2011) have shown that the skewness of the distribution of the clutches increased over the years in great tits, meaning that clutches are more concentrated in the early part of the season, while it did not change in blue tits *Cyanistes caeruleus*. By comparing these two species, Goodenough et al. (2011) showed that changes in the start and termination of the breeding season, and the distribution of clutches throughout it, could vary independently.

To the best of our knowledge, we present here the first study where all the breeding attempts during the breeding season are considered to describe the seasonal distribution of clutches and its yearly variation with ambient temperatures for a bird species. A main result is that, in spite of that the length of the breeding season has not changed significantly, we have seen that the skewness of the distribution of clutches has increased throughout the years. These results, along with the lack of variation in kurtosis, agree with those presented by Goodenough et al. (2011) for this species considering only first clutches.

## Conclusions

We conclude that (1) spring temperatures have increased by about 2 °C during the last three decades in Sagunto (Spain); (2) the breeding season of great tits has advanced by about 3 days per decade during this period; (3) in spite of this advancement, the length of the breeding season has not changed significantly, but (4) the distribution of the clutches throughout the breeding season has changed, clutches being more concentrated in the earliest part. It is therefore a good example of how changes in ambient temperatures could change the seasonal distribution of the clutches without changes in the length of the breeding season. This change in the distribution allows more pairs to lay two clutches without a significant increase in the length of the breeding season.

## Data Availability

The data presented in this study are openly available in Zenodo at DOI, 10.5281/zenodo.7418469.
